# Effects of Composition and Strength of Wheat Gluten on Starch Structure, Digestion Properties and the Underlying Mechanism

**DOI:** 10.3390/foods11213432

**Published:** 2022-10-29

**Authors:** Xiaoyang Zou, Xiaolong Wang, Liang Li, Pai Peng, Qianying Ma, Xinzhong Hu, Rudi Appels

**Affiliations:** 1 College of Food Engineering and Nutritional Science, Shaanxi Normal University, No. 620 West Chang’an Avenue, Chang’an District, Xi’an 710119, China; 2 Shaanxi Surea Group Co., Ltd., Xi’an 710003, China; 3 Faculty of Veterinary and Agricultural Sciences, University of Melbourne, Parkville, VIC 3010, Australia

**Keywords:** gluten structure, ratio of glutenin to gliadin, starch molecular order, starch digestibility

## Abstract

To understand the effect of gluten on starch digestion characteristics, the structural characteristics of protein, starch, and starch digestion attributes were explored by using flours of four wheat near-isogenic lines. Protein and starch fractions from the four flours were used to form so-called recombinant flours where glutenin and gliadin protein fractions, in different ratios, were combined with starch and heated in a water slurry at 80 °C for 5 min. We found that starch digestibility of the recombinant flours could be reproducibly modified by altering the long- and short-range molecular order of starch through varying the attributes of the gluten protein by virtue of the gluten strength as well as the proportions of glutenin and gliadins. The gluten composition changes of strong-gluten flour did not improve the starch digestion resistibility, however, for the moderate- and weak-gluten flours, the proportional increase of glutenin improved the resistance of starch to digestion through the increased long- and short-range molecular order of starch. The resistance of starch to digestion could also be enhanced with increasing gliadin, and was associated with the modified short-range molecular order of starch. We propose that flour mixtures can be optimized for specified product quality by manipulating the amounts of both gliadin and glutenin.

## 1. Introduction

Starch, as the principal storage polysaccharides in most plant-derived foods, is an important component of nutritional products that provides energy for humans and animals [1,2]. Cooked wheat starch can be quickly digested to produce large amounts of glucose [3], therefore, starch is also considered as a major glycemic carbohydrate, and the rate and extent of its digestion is a major factor in many diet-related chronic diseases [4]. In recent years, excessive intake of dietary energy has become a serious health threat, which leads to the occurrence of diseases, such as type II diabetes, hypertension, and heart disease [5]. Therefore, how to reduce the amount of digestible carbohydrates in food, decrease the digestibility of starch, and slow down the rate of postprandial blood glucose rise has become an issue to be solved.

Starch digestion is not only related to its intrinsic structure, such as the size and shape of the starch granules, the proportion of amylose and amylopectin, the chain length distribution and the crystallinity of starch [6], but also significantly affected by the external features, such as the way processing was carried out (the physical/chemical treatment of the starch, recrystallization), product type, and other components that exist in the product (protein, lipid, polyphenol) [7]. Protein, as the main component in wheat flour, has been proven to significantly influence the structure and behavior of starch under hydrothermal treatment [8], hence it is necessary to further explore the mechanisms by which the protein influences starch structural and digestive characteristics.

Glutenin and gliadin, as the main components of the gluten network in wheat-based food products, have been proven to affect starch digestion properties in food matrix through different mechanisms due to their differences in molecular weight and structural characteristics. Glutenin elasticity forms the functional basis of the gluten network, and acts as a physical barrier to retard the access/binding between starch and enzymes [9]. Moreover, the barrier effect of gluten network also impedes the water absorption of starch granules, which reduces starch hydration and subsequent gelatinization [10]. In contrast, the monomeric gliadin is easier to dissociate and combine with other substances due to its low molecular weight and high viscosity. On the one hand, the gliadin can bind to amylase and inhibit the digestion of starch [11]. On the other hand, gliadin also tends to bind with starch and block the digestion site or the pores on the surface of starch granules, thus inhibiting the entry of amylase and slowing down the enzymatic hydrolysis rate of starch [12].

It is well known that a high proportion of glutenin in wheat gluten contributes to a stronger gluten network, while the high percentage of monomeric gliadin leads to a weaker gluten with better extensibility [3,9]. As mentioned above, because the proportional increase of glutenin can improve the resistance of starch to digestion through enhanced barrier effects of gluten network, the quantitative increase of monomeric gliadin, in contrast, tends to inhibit starch digestion via the formation of starch-gliadin or amylase-gliadin complexes, it is hard to predict the trends of starch digestibility caused by the variations in gluten strength and ratio of glutenin to gliadin, and requires further study focused on the effect of gluten strength and composition on starch digestibility and the underlying mechanisms out. In this study, the effect of gluten strength and ratio of glutenin to gliadin within gluten protein on the digestive characteristics of starch were explored by using flours from four wheat high-molecular weight glutenin subunits (HMW-GSs) near isogenic lines (NILs) and their recombinant flours with different ratios of glutenin to gliadin, with the aim to clarify the complex interaction mechanism between starch and gluten protein during thermal processing and provide a reference for improving digestive resistance of starch in wheat-based foods by manipulating the composition of gluten protein.

## 2. Materials and Methods

### 2.1. Materials

As described in our previous paper [13], wheat NILs differing in HMW-GSs at *Glu*-*D1* were created by crossing Chinese winter wheat cultivar Xiaoyan-22 (N, 7 + 9, 2 + 12) and common French wheat cultivar 738 (N, 17 + 18, 5 + 10). Based on the continuous backcross and self-crossing after the initial cross, four BC_7_F_5_ NILs designated as 2001 (N/7+9/5+10), 2002 (N/7+9/2+12), 2003 (N/7+8/2+12), and 2006 (N/14+15/5+10) were developed for the separation of protein and starch. The time to forming a dough complex of the 2001, 2002, and 2003 flour samples was measured by Mixolab (Chopin Technologies, Villeneuve-la-Garenne, France) as 3.33 ± 0.18 a min, 1.74 ± 0.08 b min, 1.19 ± 0.01 c min, respectively. Therefore, we defined the flour from 2001, 2002, and 2003 as strong-strength flour, moderate-strength flour and weak-strength flour, respectively. Seeds of the four wheat NILs were milled into flour by a Brabender Quadrumat Senior mill using the AACC method 26–20.01 [14].

### 2.2. Methods

#### 2.2.1. Extraction and Separation of Gluten and Starch

Wheat flour and distilled water were mixed to form a dough and kept at room temperature for 30 min. The dough was then rinsed with distilled water until the water was clear to get the gluten. Starch was obtained by centrifugation of the collected starch water [15].

#### 2.2.2. Isolation of Gliadin and Glutenin

In order to isolate gliadin from gluten, 200 g of the gluten fractions were extracted from the 2001, 2002, or 2003 flour samples by resuspending in with 70% aqueous ethanol at a ratio of 1:15 (*w*/*v*), followed by stirring magnetically for 4 h at room temperature to fully dissolve gliadin. After centrifugation at 4000 rpm for 20 min, the collected supernatant was evaporated using a rotary evaporators (RE2000E, Shanghai Bingyue Electronic Instrument Co., Ltd., Shanghai, China) to remove most of the ethanol, and the residue was placed in an oven at 50 °C until completely dry to obtain gliadin-rich fractions. The centrifuged precipitate, after gliadin extraction, was dried to obtain glutenin-rich fractions [16]. The purity of gliadin extracts were close to 100% when determined by an automatic kieldahl apparatus (BUCHI-K375, Flawil, Switzerland) and size-exclusion high performance liquid chromatography (Thermo Scientific, Waltham, MA, USA), while the purities of glutenin extraction from 2001, 2002, and 2003 were 64%, 69%, and 76%, respectively.

#### 2.2.3. Preparation of Protein-Starch Recombinant Flours

Firstly, the gluten separated from 2001, 2002, or 2003 was mixed with starch from 2006 in the ratio of 1:5 to prepare three recombinant flours named 200115, 200215, and 200315. Secondly, the glutenin and gliadin from the gluten of each NIL were blended in the ratios of 3:1 and 1:3 to prepare two reconstituted gluten proteins, and six recoconstituted gluten proteins were obtained using the three NILs. Finally, each of the recomposed gluten protein was mixed with 2006 starch in the ratio of 1:5 to develop six recombinant flours named 200131, 200113, 200231, 200213, 200331, and 200313, respectively. The formulations of the nine recombinant flours are provided in Table 1. Thereafter, each recombinant flour was mixed with distilled water in a ratio of 1:10 (*w*/*v*) and stirred at room temperature for 20 min, immersing in boiling water on a magnetic stirrer for 5 min (internal temperature of flour-water mix 80 °C) before it was transferred to a constant temperature magnetic stirrer at 37 °C for 15 min. Finally, the sample was lyophilized, powdered, and sieved through 200 mesh.

#### 2.2.4. X-ray Diffraction and Relative Crystallinity

The crystalline properties of the recombinant flours were characterized by an X-ray diffractometer (Rigaku Dmax/2550, Shibuya, Japan) and the test parameters were set at 40 mA and 40 KV with Cu Kα radiation (λ = 1.5406 Å) [17]. The X-ray diffraction measurement was performed by scanning from 4° to 40° (2θ) at a rate of 2°/min to obtain the X-diffraction spectra. The software MDI Jade 6.0 (Materials Data Inc., Livermore, CA, USA) was used for graphic processing, and the relative crystallinity was calculated as (the areas of crystalline region/the total area of the diffractogram) × 100% [18].

#### 2.2.5. Fourier Transform Infrared (FTIR) Spectroscopy

FTIR spectra of the samples were measured using a Vertex 70 FTIR spectrometer (Bruker, Germany) equipped with the deuterated triglycine sulfate detector. All samples were mixed with potassium bromide in a ratio of 1:100 (*w*/*w*), dried and ground until the mixture was homogeneous. The spectra were obtained using a scanning band between 400 and 4000 cm^−1^ at a resolution of 4 cm^−1^. An empty cell was used as the background [19]. According to our previous study, the content of each protein secondary structure was quantified according to its specific fitting peak located at the amide I band (1600–1700 cm^−1^) [13]. Omnic (version 8.0, Thermo Nicolet Inc., Waltham, MA, USA) and Peakfit (version 4.12, SPSS Inc., Chicago, IL, USA) were used for the analyzing the amide I (1600–1700 cm^−1^) band.

#### 2.2.6. Solid State Nuclear Magnetic Resonance (NMR) Experiments

The solid-state ^13^C CP/MAS spectra of recombinant flours were recorded on a ^13^C NMR (JNM-ECZ400R/S1, Japan Electron Optics Laboratory, Akishima-shi, Japan) equipped with CP-MAS accessories. Dipolar decoupling was systematically used during the acquisition sequence. The recombinant flours sample of 200 mg was scanned at a frequency of 125.7 MHz. The optimal contact time was 13 ms, spectral width 40 kHz, Scan rate 20 Hz, Scan number 4096. PeakFit™ (Jandel Scientific Software, San Rafael, CA, USA) was used to fit characteristic peaks in the spectra the content of double helix was defined as the ratio of fitting peak area of C2,3,5 (70–75 ppm) to the total fitting peak area, the single helix proportion was defined as the ratio of the fitting peak area of V-type characteristic peak (94 ppm) to the total fitting peak area, and the content of the amorphous phase (PPA) was defined as the ratio of the fitting peak area of C4 (80–84 ppm) to the total area [20].

#### 2.2.7. Laser Confocal Micro—Roman (LCM—Raman) Spectroscopy

The short-range molecular orders of recombinant flours were obtained by using a Laser Raman spectrometer with a 785 nm green diode laser source (Renishaw, Gloucestershire, United Kingdom). Raman spectroscopy of each sample in the range of 100–3200 cm^−1^ with a resolution of approximately 7 cm^−1^ was obtained. The full width at half maximum (FWHM) of the band at 480 cm^−1^, which represented the short-range molecular order of starch, was determined by using the WIRE 2.0 software [21].

#### 2.2.8. Quantification of the Chemical Bonds in Recombinant Powder Proteins

The molecular force between gluten molecules was evaluated by the solubility of the protein in different denaturation solvents using the method described by Gómez-Guillén with some modification [22]. The solvents included 0.05 M NaCl (SA), 0.6 M NaCl (SB), 0.6 M NaCl + 1.5 M urea (SC), 0.6 M NaCl + 8 M urea (SD), and 0.6 M NaCl + 8 M urea + 0.5 M β-mercaptoethanol (SE). The recombinant flour (0.1 g) was dispersed in 5 mL of each of the above buffer solution and then centrifuged at 13,000× *g* for 20 min at room temperature. The protein concentration in supernatants was determined by the Coomassie bright blue method. The proportion of ionic bonds was determined by the content difference between proteins extracted by SB and SA, hydrogen bonds was quantified by the content difference of proteins extracted by SC and SB, hydrophobic interactions was determined by the proteins dissolved in SD and SC, and disulfide bonds was quantified according to the content difference of proteins dissolved in SE and SD.

#### 2.2.9. Detection of Intrinsic Fluorescence Spectrum

Dissolve recombinant powders (50 mg) in 15 mL PBS (0.5% SDS—0.1 M phosphate buffer, pH 7.0). Sample solutions were placed in a cuvette under temperature control at 25–50 °C. The corresponding steady-state fluorescence spectra of the samples were obtained by a fluorescence spectrometer (RF-6000, Shimadzu, Japan) upon 295 nm excitation at a spectral resolution of 2.5 nm, and emission spectrograms between 300 and 400 nm were acquired at a rate of 100 nm/min [23].

#### 2.2.10. Size-Exclusion High Performance Liquid Chromatography (SE-HPLC) Analysis of Recombinant Powder Proteins

According to previous research with minor modifications [13], the apparent molecular weight distribution of recombinant flours proteins was evaluated by an Ultimate 3000 HPLC system (Thermo Scitific, Waltham, USA) with a TSK gel G4000SWXL (7.8 × 300 mm) column (Tosoh Corporation, Tokyo, Japan). Samples (50 mg) were suspended in 5 mL of 0.05 M phosphate buffer solution (containing 0.5% SDS, pH 7.0), in order to extract SDS-extractable proteins. SDS-unextractable proteins were then separated from the remaining residue by ultrasonic treatment (VS-2100UE, Wuxi Woxin, China) (ultrasonic 2 sec followed by static 2 s as one cycle, for 2 min). The sample (20 μL) was automatically injected into the column with the PBS and allowed to run for 25 min. With the decrease of molecular weight, the five parts of the protein extraction chromatogram are, respectively, represented as the largest polymeric protein (LPP), the middle polymeric protein (MPP), the smaller polymeric protein (SPP), and the larger and smaller monomeric protein (LMP and SMP). The proportion of SDS-unextractable LPP and MPP was designated as UPP%. The protein content was expressed as the peak area of each part divided by the total area. The peaks of SDS extractable and unextractable LPP, MPP, SPP, LMP, and SMP were named F1e and F1u, F2e and F2u, F3e and F3u, F4e and F4u, F5e and F5u, respectively. The contents of different protein components were calculated as follows:

Total area = F1e + F1u + F2e + F2u + F3e + F3u + F4e + F4u + F5e + F5u(1)

LPP% = 100 × (F1e + F1u)/Total area(2)

MPP% = 100 × (F2e + F2u)/Total area(3)

SPP% = 100 × (F3e + F3u)/Total area(4)

LMP% = 100 × (F4e + F4u)/Total area(5)

SMP% = 100 × (F5e + F5u)/Total area(6)

UPP% = 100 × (F1u + F2u)/Total area(7)

#### 2.2.11. In Vitro Digestibility of Recombinant Flours

In vitro digestibility of recombinant flours was tested according to an existed method with a slight modification [24]. Samples (containing 50 mg starch) were weighted into a 50 mL reaction flask containing 10 mL acetate buffer (0.5 M, pH 5.2) and four glass balls, and reaction flasks were shaken at 37 °C for 15 min. Following the shaking process, 5 mL mixed solution of enzyme containing 4 mL of 30 mg/mL porcine pancreatic enzyme solution (4 × USP, P1750—100 g, Shanghai Hengdu Biotechnology Co., Ltd., Shanghai, China) and 1 mL of 2500 U/mL glucoamylase solution (1 × 10^5^ U/mL, A107823—10 mL, Shanghai Aladdin Biochemical Technology Co., Ltd., Shanghai, China) were transferred into each reaction flask and incubated in a 37 °C water bath for 180 min with constant shaking (120 rpm). Hydrolyzed solution (1 mL) was withdrawn at specific time points (0, 10, 20, 30, 60, 90, 120, 180 min), then boiled for 5 min to stop the enzyme reaction. The solution was centrifuged at 4000 rpm for 10 min and, after proper dilution, the glucose concentration in supernatant was measured by a biosensor (S-10, Shenzhen Sieman Technology Co., Ltd., Shenzhen, China) after calibrating with a standard glucose solution of 1 mg/mL. Digestibility and contents of rapidly digestible starch (RDS), slowly digestible starch (SDS) and resistant starch (RS) were calculated by the following formulas:

(8) $$A\%=\left[\frac{C\times V\times n}{TS}\right]\times 0.9\times 100$$

(9) $$\mathrm{RDS}\%=\left[\frac{G20-NG}{TS}\right]\times 0.9\times 100$$

(10) $$\mathrm{SDS}\%=\left[\frac{G120-G20}{TS}\right]\times 0.9\times 100$$

(11) $$\mathrm{RS}\%=\left[\frac{TS-RDS-SDS}{TS}\right]\times 100$$

where A% is the digestibility; C is glucose concentration, mg/mL; V is the volume of liquid in the reaction system, mL; n is diluted multiples; *TS* is the total starch quality, mg; NG is the content of native glucose in starch; G_20_ and G_120_ are the glucose content respectively released within 20 min and 120 min of hydrolysis, mg.

#### 2.2.12. Statistical Analysis

All tests were performed in triplets unless stated otherwise, and the results were presented as mean ± standard deviation. The one way analysis of variance (ANOVA) was processed by SPSS statistical software (19.0, SPSS Inc., Chicago, IL, USA) with the Duncan’s test at a significance level of 0.05. A correlation analysis between starch digestibility and the structural characteristics of protein and starch was performed using an Originpro Learning Edition. The Spearman correlation coefficient was employed to represent the degree of each correlation.

## 3. Results and Discussion

### 3.1. In Vitro Digestibility of Recombinant Flours

**Figure 1:**
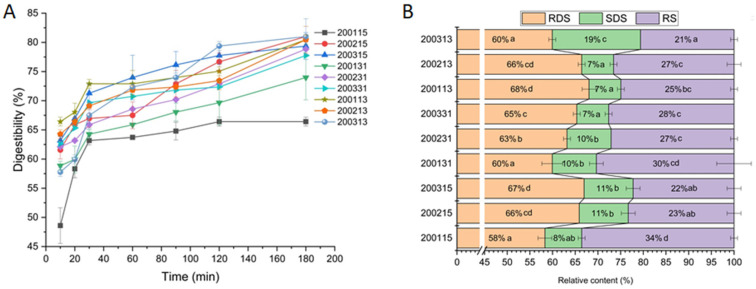
In vitro digestion curve (**A**) and the contents of RDS, SDS, and RS (**B**) in recombinant flours. (Means with different small letters within the same category are significantly different at *p* < 0.05).

The in vitro digestion curve of all recombinant flours showed faster digestion rates of starch within the first 30 min (Figure 1). Comparing 200215 and 200315, significantly more RS but less RDS and SDS were found in 200115, indicating that stronger gluten strength could reduce the digestibility of starch. There were no significant differences in RDS, SDS, and RS between 200215 and 200315, indicating that the difference in HMW-GSs at *Glu*-*B1* had little if any effect on starch digestion. For the 2001 samples, the proportional increase of glutenin in gluten protein (200131) had no significant effect on the content of RDS, SDS, and RS, however, a significant content decrease in RS and increase in RDS were found in the gluten containing more gliadin (200113). This difference in 2001 samples indicated that the barrier effect of strong-strength gluten protein on starch granules was weakened with the increasing proportion of gliadin, and the inhibiting effect of macromolecular protein network on starch digestibility was greater than that of monomeric protein. For 2002 samples, part of the RDS was converted to RS after the increase of glutenin (200231), while part of SDS was converted to RS after the increase of gliadin (200213). The difference between 200231 and 200213 indicated that for moderate-strength gluten protein, the enhanced coating effect of the stronger gluten network mainly inhibited the rapid digestion of starch, while the additional monomeric proteins were more prone to interacting with amylase or starch and reducing the amount of SDS. Although there was no difference in RS content between 200231 and 200213, more SDS and less RDS were detected in 200231 than those in 200213, which indicated that for the flour with moderate-strength gluten, compared with the proportional increase of monomeric gliadins, the improved strength of gluten containing more glutenins was more conducive to reducing the starch digestibility. For 2003 samples, part of RDS and SDS were converted into RS after the increase of glutenin, and part of RDS was converted to SDS with the increase of gliadin, which indicated that for the flour with weak-strength gluten, the enhancement of gluten strength through quantitative increase of glutenin was more effective to inhibit starch digestion than the proportional increase of monomeric gliadins. In brief, for the flour with moderate or weak-gluten strength, the proportional increase in glutenin and gliadin significantly decreased the starch digestibility. The inhibition effect of an enhanced macromolecular gluten network on starch digestion was stronger than that of gluten protein with more monomeric proteins. However, for the flour with high-strength gluten protein, further enhancement of macromolecular gluten network or incorporation of additional monomeric gliadins did not improve the resistance of starch to digestion.

### 3.2. The Advanced Structure of Proteins in the Recombinant Flours

**Figure 2:**
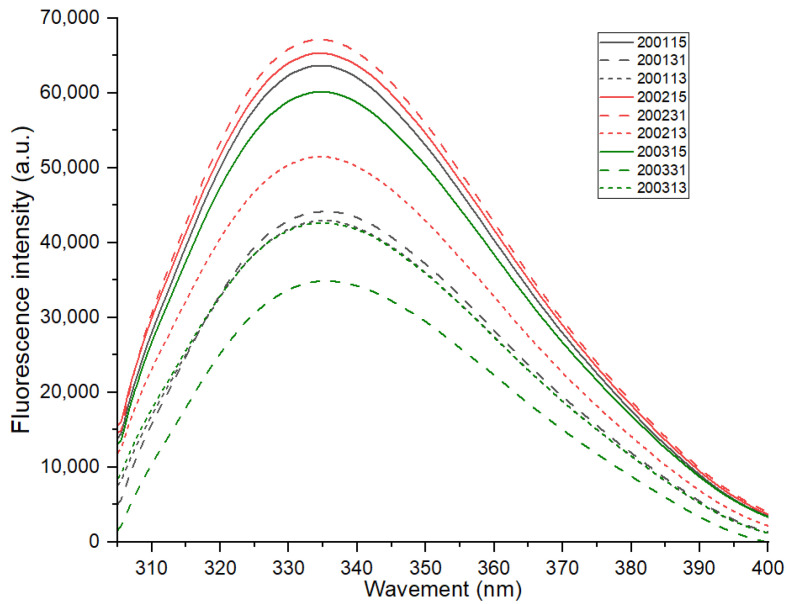
Fluorescence spectra of the recombinant flours.

The maximum fluorescence emission of proteins (about 337 nm) varies with the polarity of tryptophan residues in the environment [15]. As shown in Figure 2, there was no significant difference in the maximum fluorescence emission of all samples, indicating no significant differences in the environmental polarity of tryptophan in different recombinant flours. The interaction between the chromogenic residues and the surrounding groups will change the frequency and intensity of the fluorescence spectrum [25]. The covalent or non-covalent combination of the chromogenic group with non-chromogenic group will form a strong fluorescence complex [26]. For all the samples, their fluorescence intensity decreased to varying degrees with the proportional increase of gliadin, and this may be related to the higher quantity of tryptophan in glutenin than that in gliadin [27]. In addition, except for the recombinant flours 200113 and 200313 which showed nearly overlapped florescence spectra, the fluorescence intensity of the recombinant flours with the same glutenin-gliadin ratio was always ranked as 2002 > 2001 > 2003, and when the proportion of glutenin was increased, the difference in gluten strength resulted in a greater change in fluorescence intensity. These results indicated that, a quantitative increase of glutenin in moderate-strength gluten (200231) was more likely to promote the aggregation of gluten protein, while glutenin content increase in strong-strength gluten (200131) tend to fracture gluten network during cooking due to excessive gluten strength.

### 3.3. Protein Behaviors of the Recombinant Flours

According to a previous study [8], the polymeric proteins (LPP, MPP, SPP) in the recombinant flour are mainly composed of glutenin and gliadin, while monomeric gliadin is the main component of LMP, and SMP is mainly composed of albumin and globulin. As shown in Table 2, in the recombinant flours prepared with starch and different natural gluten proteins from the three wheat NILs (200115, 200215, 200315), no significant difference in the contents of polymeric and monomeric proteins among the three samples were found, but the UPP% in 200115 was significantly higher than that in 200215 and 200315, which was consistent with the strong-strength gluten contributing to the formation of thermally stable protein polymers remained intact structure during cooking. For the wheat NILs of 2001 and 2003, the proportional increase of glutenin or gliadin significantly decreased the contents of LPP, MPP and UPP, and the high proportion of gliadin in gluten protein (200113 and 200313) led to greater reduction in LPP, MPP, and UPP compared to the gluten proteins with high percentage of glutenin (200131 and 200331), in contrast, the content of SPP and LMP increased significantly with the ratio variation of glutenin to gliadin, and the highest content of SPP or LMP was found in gluten with high percentage of gliadin (200113 and 200313). All these results showed that, for the flours with strong and weak-strength gluten proteins, the modification of glutenin-gliadin ratio (approximately 1:1 in natural gluten protein) led to a trend for damaging the stability and strength of the gluten networks. This tendency for reduced stability and strength of the gluten networks was attributed to the fact that the LPP, MPP, and UPP were partially depolymerized into SPP and LMP. Furthermore, compared with the gluten protein with high content of glutenin (200131, 200331), the proportional increase of gliadin caused much greater damage to gluten network. Different from 200131 and 200331, 200231 had the highest content of LPP, MPP, UPP, and the lowest content of LMP among all the recombinant flours. Therefore, we believe that the quantitative increase of glutenin in moderate-strength gluten is more conducive to the formation of a much stronger gluten network, and also promoted the binding of monomeric gliadin to amylose or short amylopectin, all these changes consequently led to the low content of RDS (Figure 1B). Within each group of recombinant flours, the content of monomeric gliadin (LMP) was lowest when natural gluten was recombined with starch, probably because the separation of glutenin and gliadin disrupted the original protein network.

### 3.4. Comparison of Protein Secondary Structure of the Recombinant Flours

For the three wheat NILs, significant differences in the contents of α--helix and β-sheet under different glutenin-gliadin ratios were found (Table 3). Quantitative increase of gliadin in the gluten protein of the three NILs always led to increased content of α-helix, which was consistent with the finding that gliadin was rich in α-helix [28]. The change of α-helix content was opposite to the change of UPP% (Table 2) in each group of the recombinant flours, which indicated that the quantity of α-helix was negatively correlated with the gluten strength during heat treatment. This assertion is supported by our previous finding that a lower content of α-helix may be attributed to the intermolecular disulfide bonds breaking and gluten rearrangement induced by heating treatment [8]. The content of β-sheet was positively correlated with the stability of protein secondary structure [8]. The increase in glutenin led to increased β-sheet content in the recombinant flours, which confirmed the report that glutenin contained higher content of β-sheet than that of gliadin [29]. To further explore the potential relationship between the secondary structure characteristics and protein polymerization/depolymerization behavior in recombinant flours, we calculated the ratio of β-sheet to α-helix (β-sheet/α-helix). It can be found that when the β-sheet/α-helix was lower than 1.7, it has a linear, positive, correlation with the UPP% (Table 2) which indicated that within a certain range the higher β-sheet/α-helix can be used to characterize the formation of strong and thermally stable gluten network. The same variation in the trend of disulfide bond and β-sheet/α-helix formation in the recombinant flours (Table 3 and Table 4) was consistent with this suggestion.

For the recombinant flours containing natural gluten protein and starch, 200115 had the optimal β-sheet/α-helix compared to 200215 and 200315, which contributed to the formation of more compact and stable gluten network that inhibited the enzymatic hydrolysis of starch and produced more RS and less RDS (Figure 1B). For the recombinant flours containing higher content of glutenin, excessively high β-sheet/α-helix in 200131 increased the digestibility of starch slightly, although the RDS’s in 200231 and 200331 were still higher than in 200131, consistent with the increased β-sheet/α-helix content improving the starch digestion resistibility as reflected in their significantly increased RS (Figure 1B). The increase of gliadin in gluten protein caused a significant decrease of β-sheet/α-helix in 200113 and 200213. The β-sheet/α-helix decrease in 200113 led to weakened gluten network, thus resulting in increased starch digestibility characterized by significant increase in RDS and decrease in RS compared to 200115. However, the reduced β-sheet/α-helix in 200213 caused decrease in SDS and increase in RS, indicating that additional gliadins added in moderate-strength gluten tended to enhance the interactions between monomeric gliadin and starch or amylase, thus converting part of SDS to RS. In 200313, the high proportion of gliadin led to significant increases in α-helix and β-sheet when compared to 200315, but no significant change in β-sheet/α-helix was found. The additional gliadins in weaker gluten protein (200313) appeared to convert partial RDS (from 67% to 60%) into SDS (from 11% to 19%) through the enhanced interactions between monomeric proteins and starch or amylase.

### 3.5. Quantitative Changes of Different Chemical Bonds within Protein of the Recombinant Flours

As a relatively stable covalent bonds, disulfide bonds play an important role in stabilizing the spatial structure of the peptide chain. As shown in Table 4, under the same glutenin-gliadin ratio, the content of disulfide bond in recombinant flours was always ranked as 2001 > 2003 > 2002. According to the higher UPP% and β-sheet/α-helix in 2001 samples (Table 2 and Table 3), it is evident that the more sulfhydryl-disulfide bond exchange reaction and sulfhydryl oxidation occurred in the cooking of the recombinant flours from 2001 promoted the formation of a more extensive and compact gluten network [30]. The content of disulfide bond in 2003 samples was higher than that in 2002 samples, which might be due to the greater contribution of subunits 7 + 8 than subunits 7 + 9 to the accumulation of gluten proteins in wheat kernel [31]. In general, the disulfide bond content increased with the increase of glutenin-gliadin ratio in all the recombinant flours. This is consistent with the report that the disulfide bonds in gluten network are mainly formed through the oxidative crosslinking of cysteine residues located at the N- or C-terminal of HMW-GSs and LMW-GSs [32], while fewer disulfide bonds between monomeric gliadin and glutenin were generated when the temperature was higher than 80 °C [33]. The significantly lower quantity of disulfide bonds in the gluten proteins with increased gliadin further showed that the additional gliadins in gluten protein interrupted the formation of disulfide bonds. Notably, more disulfide bonds were detected in 200331 compared to 200315, but the content of polymeric proteins and UPP% in 200331 were significantly lower than those in 200315 (Table 2), this contradiction implied that more intramolecular disulfide bonds or intermolecular disulfide bonds between glutenin and gliadin were formed in 200331, and impeded the development of extensive gluten networks. In contrast, 200231 had the highest UPP% in all the recombinant flours, but a lower quantity of disulfide bonds was detected. Considering the strongest tryptophan fluorescence intensity of 200231 among all the recombinant flours (Figure 2), we suppose that a strong protein-starch interaction has occurred in 200231, and reduced the protein extractability significantly.

Hydrophobic interactions are the main driving force of protein folding. For the three NILs, the intensity of hydrophobic interaction was increased to varying degrees with the increase of glutenin content, indicating that higher glutenin content could promote the aggregation of hydrophobic residues and stabilize the structure of protein networks. For 2001 samples, the hydrophobic interaction was always weaker than most of the 2002 and 2003 samples, which may be attributed to the stronger protein-starch interaction in 2001 samples that hindered the aggregation of hydrophobic groups in protein. This prediction was also confirmed by the significantly higher hydrogen bond content in 2001 samples [8]. Notably, protein in 200313 was characterized by weak hydrophobic interactions but high content of hydrogen bonds, indicating the relatively strong protein-starch interaction that resulted low content of RDS in 200313 (Figure 1B).

The recombinant flours directly prepared by natural gluten and starch showed much higher content of hydrogen bonds compared to the flours with increased proportion of glutenin or gliadin, this difference may be due to the fact that some original hydrogen bonds existing in the gluten extractions were broken in the separation of glutenin and gliadin. Furthermore, when the proportion of glutenin or gliadin was increased, the hydrogen bond content decreased to different degrees and the lower content of hydrogen bond was accompanied by the higher content of gliadin. This phenomenon indicated that the incorporation of additional glutenin or gliadin led to the rearrangement of original gluten structure. As for the gluten with increased content of glutenin, the decreased hydrogen bond was accompanied by the significant increases in both hydrophobic interactions and disulfide bonds, resulting in a more extensive and stable gluten network, however, for the gluten with increased gliadins, significant decreases in disulfide bonds and hydrogen bonds indicated the destruction of gluten structure. The content of ionic bonds in gluten protein was significantly lower compared with the other chemical bonds. Moreover, regardless of the composition of gluten protein, the flours containing 5 + 10 subunits showed relatively high ionic bonds, which was consistent with our previous study [8].

### 3.6. Long- and Short-Range Molecular Order of Starch in Recombinant Flours

**Figure 3:**
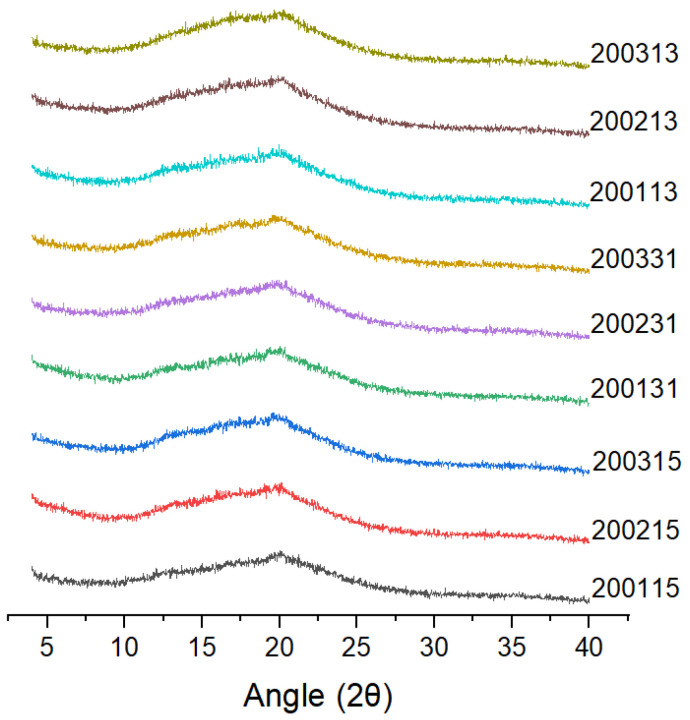
X-ray diffraction patterns of the recombinant flours.

X-ray diffraction (Figure 3) showed that all the cooked recombinant flours showed typical V-shaped crystallization peaks with a strong diffraction peak at 19.95°, which was mainly due to the combination of amylose and a series of micromolecules (such as lipids or proteins) to form a single left--handed complexes [17].

**Figure 4:**
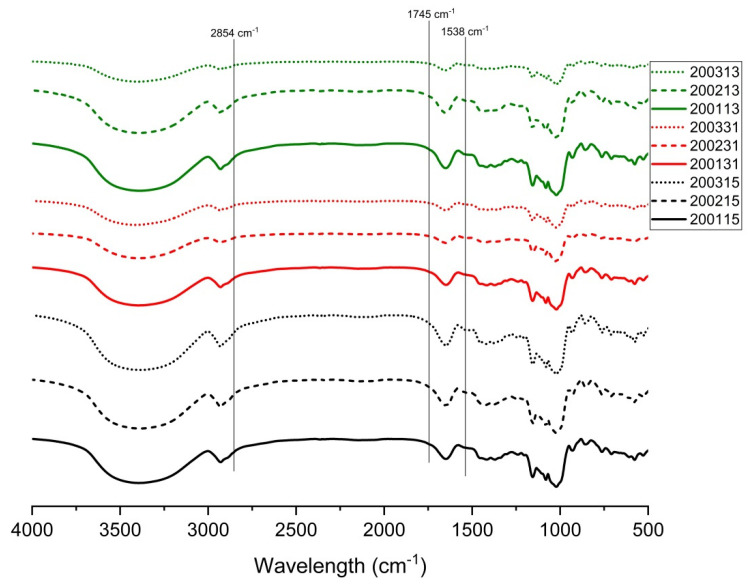
FTIR transmittance spectra of starch in recombinant flours.

In the FTIR spectrum, the peak at 1538 cm^−1^ was correlated with the presence of starch-protein complex, the peaks at 1714 cm^−1^ and 2854 cm^−1^ indicated the existence of lipid-starch complex, and the simultaneous appearance of the three peaks indicated the presence of starch-lipid-protein complexes in the samples [34]. As shown in Figure 4, all the cooked recombinant flours showed peaks with different intensities at 1538 cm^−1^, indicating the formation of starch-protein complexes. No obvious peak was observed at 2854 cm^−1^ and 1745 cm^−1^, which possibly indicated that the low content of lipid in wheat flour and the further lipid loss during separating of gluten proteins and starch reduced the possibility for forming starch-lipid or starch-lipid-protein complexes. Compared with other flours, more obvious peaks at 1538 cm^−1^ were observed in 200115, 200215, 200315, 200113, and 200231, in view of their lower LMP contents (Table 2), we proposed that more starch-protein complexes were formed in these flours.

For all the recombinant flours, the relative crystallinity of starch increased significantly with the increase of glutenin content, while the high content of gliadin led to the significant reduction in relative crystallinity (Table 5). This result indicated that the high proportion of glutenin contributed to a more extensive and compact gluten network that effectively prevented the swelling and gelatinization of starch, while additional monomeric gliadins had negative effect to the gluten strength and long-range molecular order of starch. Notably, the recombinant flours with relatively stronger gluten strength, such as 200115, 200231, and 200331, showed comparatively higher relative crystallinity that supposed to enhance the starch digestion resistibility and produce more RS in starch digestion (Figure 1B).

In the FTIR spectrum, the absorbance at 995 cm^−1^ and 1047 cm^−1^ is related to the order degree of starch molecules, while the sensitivity at 1022 cm^−1^ is linked to the amorphous structure of starch. Therefore, the degree of orderliness (DO) and the degree of double helix (DD) of starch can be characterized by 1047/1022 and 995/1022, respectively [35]. Moreover, the full width at half-maximum (FWHM) of the band at 480 cm^−1^ of LCM-Raman spectrum was also used to characterize the short-range molecular order of starch [36]. As shown in Table 5, the starch in 200215 and 200315 showed higher DO and DD values and lower FWHM than the other recombinant flours, indicating the natural moderate- or weak-strength gluten protein containing approximately equal amount of glutenin and gliadin could promote the formation of more ordered starch structure rich in double helices. For the 2001 samples, high proportion of gliadin in gluten was contributed to the high short-range molecular order of starch in 200113 that characterized by higher DO, DD, and lower FWHM. This result can be interpreted in two ways: firstly, the weak-strength gluten network of 200113 facilitated the pasting of starch, thus releasing more short-branch starch that incorporated into the formation of more double helix; secondly, additional monomeric gliadins enhanced the interaction between gliadin and short-chain starch, and eventually improved the short-range molecular order of starch. Notably, within the three recombinant flours prepared by natural gluten and starch (200115, 200215, 200315), the decrease in FWHM and increases in DO and DD were observed with the descend of the gluten strength, which may be due to the fact that the weak gluten network was more likely to break and depolymerized during cooking, thus facilitating the interactions between protein and starch fragments and improving the short-range molecular order of starch. The DO or DD of 200113 and 200213 was significantly higher than that of 200131 and 200231, respectively, which indicated that the incorporation of additional gliadins probably improved the short-range molecular order of starch in strong- and moderate-strength gluten flour by enhancing the interactions between monomeric gliadins and gelatinized starch.

Besides the non-covalent bonding of protein to starch, the covalent interaction between protein and starch was detected according to the shifts of the bands at ~860 cm^−1^ that were sensitive to the anomeric structure around glycosidic bonds [15]. The peak wavelength and transmittance changes of the recombinant flours were accompanied by the variation in glutenin-gliadin ratio (Table 5), suggesting gluten strength and the ratio of glutenin to gliadin could significantly affect the binding between monomeric protein and starch. Notably, a peak wavelength migration and a relatively lower transmittances were found in the recombinant flours composed by natural gluten proteins and starch, indicating that stronger glycoside bonds between protein and starch were existed in these flours. The low amount of LMP in 200115, 200215 and 200315 (Table 2) was consistent with this judgment. The decrease of gluten strength, related to a significant decreased in transmittances, which may be due to the fact that the protein network in 200315 was more damaged during cooking, thus forming stronger glycosidic bonds between gliadin and amylose/amylopectin. For the recombinant flours with high content of glutenin (200131, 200231, 200331), the peak deviation was only found in the weak-gluten flour (200331), which indicated the existence of strong protein-starch interaction. Although there was no change of wavenumber in 200131 and 200231, the transmittance in 200131 was significantly lower than that in 200231, indicating that the starch in the strong-gluten flour was less damaged and retained more glycoside bonds. For the recombinant flours containing high content of gliadin (200113, 200213, 200313), the shift extent of peak and transmittance were increased with the decrease of gluten strength, indicating weaker glycosidic linkage between monomeric gliadin and starch was more prone to from in the flours with weak-strength gluten.

**Figure 5:**
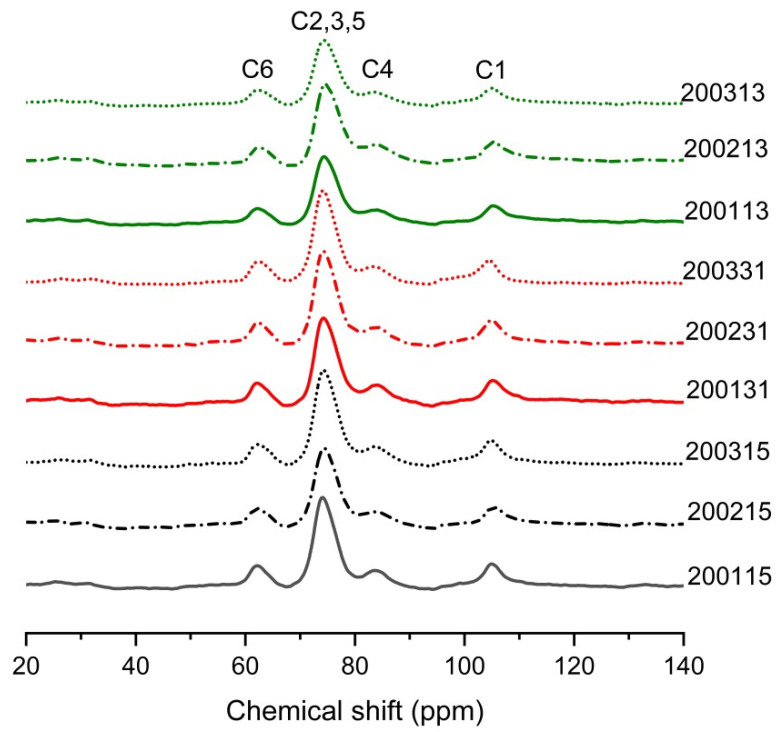
The ^13^C solid state NMR spectra of different recombinant flours.

NMR can be used to assess helix content in starch [37]. The wide peak of C1 at 103 or 104 ppm corresponded to typical single helices organized as V-type crystalline phases or dispersed in amorphous phases, while the peak near 76 ppm was positively correlated with the content of double helix in the starch of the recombinant flours [38]. As shown in Figure 5, the V-type crystals formed in all recombinant flours. As shown in Table 6, for 2002 and 2003 samples, an increase in content of single and double helices was observed after the quantitative increase of glutenin, suggesting the introduction of additional glutenin in moderate- and weak-strength gluten flours promoted the formation of stable protein network, thus reducing the degree of starch fragmentation during high-temperature cooking [13]. For 2001 samples, the single helix was the highest in 200115, but the content of the double helix was the lowest, we assumed that the cooking promoted the unwinding of amylose/short amylopectin and facilitated the formation of more starch-lipid/protein complex [39]. The lower LMP% in 200115 compared to 200131 and 200113 confirmed this indication of protein-starch complex formation (Table 2). In addition, the lipid loss during the preparation of recombinant flours with high content of glutenin or gliadin may be impeded by the formation of V-type starch-lipid complex in 200131 and 200113, thus further increasing the content of single helix in 200115. For the weak-strength gluten flours, the content of PPA decreased after the proportion of glutenin or gliadin increased, which was consistent with the finding that the content of RDS in 200331 and 200313 was significantly lower than that in 200315 (Figure 1B).

Combining the single helix content in the starch from different recombinant flours and the shift and intensity of the 860 cm^−1^ peak from FTIR, we inferred that the covalent protein-starch complex accounted for a high proportion of the single helix in the recombinant flours containing natural gluten. In contrast, more non-covalent protein/lipid-starch complexes may be involved in the high content of single helix in 200231 and 200331. For the recombinant flours with increased gliadin, there were still more starch fragments linked to the protein (Figure 4 and Table 5) although the single helix was damaged greatly due to the decrease of the gluten strength.

### 3.7. Correlationship between Starch Digestibility and the Structal Characteristics of Protein and Starch

**Figure 6:**
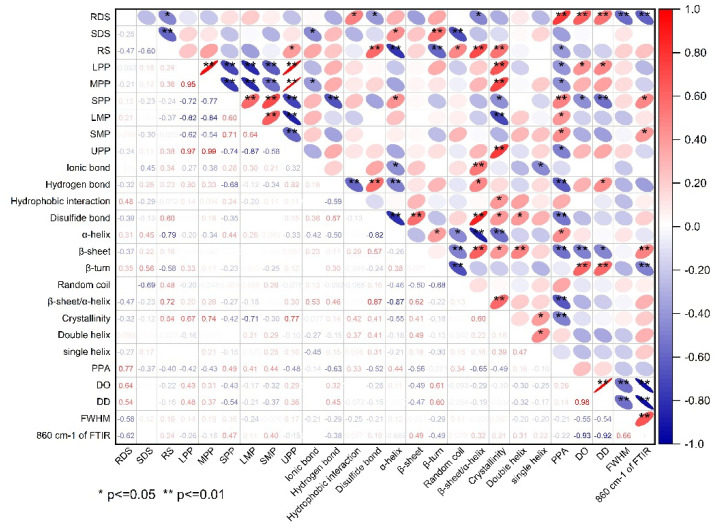
The correlation between starch digestiblity and structural characteristics of protein and starch. The red and blue ellipses indicated positive and negative correlations, respectively, and the degree of correlation was increased with decreased area and increased color depth of the ellipses. The red-blue ruler on the right of the correlation matrix illustrated the correspondense between color and correlation coefficient.

Correlation analysis between starch digestibility and the structural changes of protein and starch is shown in Figure 6. It can be found that the content of RS was significantly positively correlated with disulfide bonds, β-sheet/α-helix, relative crystallinity of starch, and the content of UPP. In contrast, it was significantly negative corrlated to α-helix, β-turn, and PPA of starch. These findings reconfirmed that an extensive and compact gluten network rich in disulfide bonds and high β-sheet/α-helix ratio can inhibite the gelatinization and digestion of starch granules, thus resuting in a high content of RS. There was an extremely negative correlation between the content of SDS and RS, and the content of SDS was significant positively related to α-helix and β-turn, but significanlty negatively related to ionic bonds and random coil. These results indicated that a relatively compact gluten strength contributed to the formation of SDS and the weakening of th gluten network properly promoted the conversion of RS to SDS. The content of RDS was significant positivley reated to hydrophobic interactions of protien, but significantly negatively related to disulfide bonds and β-sheet/α-helix, as this phenomonon explained the fact that the unfolding of gluten network wrapping around the starch granules significantly facilitated the gelatinization and digestion of starch, leading to a high proportion of RDS in starch. Moreover, the content of RDS was positively related to PPA, DO, and DD values, but negatively related to FWHM and the peak transmittance at ~860 cm^−1^ FTIR, which revealed that the covalent starch-potein complexes formed in amorphous region tended to inhibit the rapid digestion of starch, however, the high short-range molecular order of starch probably accelerated the digestion of starch. All the results above indicated that the compostion and strength of gluten protein can significantly affect the structual characteristics of gluten proteins as relfected by their variations in chemical bonds, secondary structure, and degree of polymerization. The differences in gluten structure further led to significant changes in protein-starch interactions and the long- and short molecualr order of starch, which finally determined the gelatinization and digestion of starch in different component flours.

## 4. Conclusions

The ratio of glutenin to gliadin in gluten had different effects on the digestive characteristics of the flours from NILs with different gluten strength. For the flour with strong-strength gluten, the increase of glutenin content led to an excessively strong but fragile gluten that resulted in the damage of the long-range molecular order (relative crystallinity). Improvement of the short-range molecular order (FWHM, double helix content) of starch, ultimately showed an insignificant influence on starch digestive characteristics. We note that when the proportion of gliadin increased, the short-range order of starch (DO, DD value and double helix, FWHM) was improved, although the relative crystallinity of starch decreased significantly due to the reduced strength and stability of gluten network, which ultimately promoted the conversion of RS to RDS. For the flours with moderate-strength gluten, the increase of glutenin content improved the degree of polymerization within the gluten network, which increased the relative crystallinity and content of single/double helix in starch and converted some RDS into RS. However, the proportional increase of gliadin led to the increased content of double helix in starch although the long-and short-range molecular order of starch was decreased. This change promoted the conversion from SDS to RS. For the flours with weak-strength gluten, the increase of glutenin content made up for the lack of gluten network, thus improving the relative crystallinity and single helix content of starch and facilitating the conversion of RDS and SDS to RS. In contrast, the increase of gliadin content decreased the gluten compactness, but led to more glycosidic linkages between monomeric gliadin and starch although both the long- and short-range molecular order of starch were decreased, which ultimately resulted in the partial transformation of RDS into SDS. Generally, the glutenin in gluten mainly affected the long-range order of starch, while the short-range order of starch was affected by both glutenin and gliadin. This study has clarified the mechanism by which the composition and strength of gluten protein can affect starch digestibility and provide a model for developing consumable low-GI foods at a broader level.

## Figures and Tables

**Table 1:** The formulation of each recombinant flour.

Sample	Protein	Starch
200115	16.67% gluten from 2001 flour	83.33% starch from 2006 flour
200215	16.67% gluten from 2002 flour	83.33% starch from 2006 flour
200315	16.67% gluten from 2003 flour	83.33% starch from 2006 flour
200131	12.50% glutenin, 4.17% gliadin from 2001 flour	83.33% starch from 2006 flour
200231	12.50% glutenin, 4.17% gliadin from 2002 flour	83.33% starch from 2006 flour
200331	12.50% glutenin, 4.17% gliadin from 2003 flour	83.33% starch from 2006 flour
200113	4.17% glutenin, 12.50% gliadin from 2001 flour	83.33% starch from 2006 flour
200213	4.17% glutenin, 12.50% gliadin from 2002 flour	83.33% starch from 2006 flour
200313	4.17% glutenin, 12.50% gliadin from 2003 flour	83.33% starch from 2006 flour

**Table 2:** Relative content of different protein fractions in the recombinant flours.

Sample	LPP (%)	MPP (%)	SPP (%)	LMP (%)	SMP (%)	UPP (%)
200115	10.45 ± 0.51 ef	24.91 ± 0.57 g	10.19 ± 0.31 a	50.99 ± 0.14 b	3.46 ± 0.22 ab	27.44 ± 0.17 h
200215	10.92 ± 0.29 f	22.65 ± 0.38 f	11.71 ± 0.06 b	52.02 ± 1.11 bc	2.70 ± 1.15 a	20.36 ± 0.02 g
200315	9.99 ± 0.11 e	23.01 ± 0.32 f	10.32 ± 0.21 a	52.99 ± 0.12 c	3.68 ± 0.11 abc	19.82 ± 0.05 f
200131	7.14 ± 0.07 d	18.47 ± 0.04 e	11.66 ± 0.54 b	59.03 ± 0.12 d	3.71 ± 0.45 abc	16.19 ± 0.22 e
200231	13.75 ± 0.46 g	27.24 ± 0.35 h	11.63 ± 0.03 b	43.99 ± 0.05 a	3.39 ± 0.12 ab	30.56 ± 0.01 i
200331	2.39 ± 0.15 b	15.87 ± 0.35 c	14.02 ± 0.51 d	62.84 ± 0.32 e	4.88 ± 0.32 c	7.87 ± 0.10 c
200113	2.78 ± 0.01 b	13.60 ± 0.35 b	12.60 ± 0.20 c	67.07 ± 0.69 f	3.96 ± 0.84 abc	5.53 ± 0.11 b
200213	5.86 ± 0.24 c	17.37 ± 0.03 d	14.08 ± 0.23 d	58.53 ± 0.47 d	4.15 ± 0.52 bc	12.66 ± 0.13 d
200313	1.18 ± 0.01 a	10.92 ± 0.38 a	14.26 ± 0.29 d	69.48 ± 0.27 g	4.18 ± 0.17 bc	3.25 ± 0.02 a

The different lowercase letters associated with the values within the same column indicate significant difference at the level of *p* < 0.05.

**Table 3:** Proportions of different protein secondary structure types in different recombinant flours.

Sample	α-Helix (%)	β-Sheet (%)	β-Turn (%)	Random Coil (%)	β-Sheet/α-Helix
200115	9.78 ± 0.05 a	16.95 ± 0.11 b	46.13 ± 0.04 b	27.15 ± 0.12 f	1.73 ± 0.03 e
200215	18.28 ± 1.20 ef	21.57 ± 2.37 c	53.21 ± 3.44 c	6.94 ± 0.14 a	1.18 ± 0.02 c
200315	16.68 ± 0.21 d	17.64 ± 0.61 b	48.91 ± 0.52 b	16.76 ± 0.31 d	1.06 ± 0.03 b
200131	13.47 ± 0.14 b	31.03 ± 0.06 e	47.76 ± 0.19 b	8.01 ± 0.12 a	2.30 ± 0.05 f
200231	14.78 ± 0.11 c	23.88 ± 0.03 d	47.20 ± 0.15 b	14.60 ± 0.58 c	1.62 ± 0.03 d
200331	13.01 ± 0.90 b	24.11 ± 0.61 d	42.24 ± 3.06 a	20.64 ± 2.77 e	1.85 ± 0.12 e
200113	14.59 ± 1.32 c	17.44 ± 0.91 b	47.94 ± 0.71 b	20.02 ± 1.52 e	1.20 ± 0.02 c
200213	19.26 ± 0.07 fg	14.37 ± 0.04 a	46.47 ± 0.07 b	19.90 ± 0.04 e	0.75 ± 0.05 a
200313	20.30 ± 0.07 g	20.99 ± 0.01 c	47.25 ± 0.57 b	11.06 ± 0.08 b	1.03 ± 0.02 b

The different lowercase letters associated with the values within the same column indicate significant difference at the level of *p* < 0.05.

**Table 4:** Quantitative changes of different chemical bonds in different recombinant flours.

Sample	Ionic Bond (mg/mL)	Hydrogen Bond (mg/mL)	Hydrophobic Interaction (mg/mL)	Disulfide Bond (mg/mL)
200115	0.0020 ± 0.0002 c	0.0170 ± 0.0005 g	0.0175 ± 0.0004 a	0.0174 ± 0.0006 e
200215	0.0027 ± 0.0004 d	0.0083 ± 0.0001 e	0.0268 ± 0.0003 d	0.0098 ± 0.0002 b
200315	0.0008 ± 0.0001 a	0.0084 ± 0.0001 e	0.0245 ± 0.0001 c	0.0109 ± 0.0002 b
200131	0.0047 ± 0.0001 e	0.0153 ± 0.0002 f	0.0222 ± 0.0004 b	0.0357 ± 0.0011 g
200231	0.0015 ± 0.0002 bc	0.0038 ± 0.0003 b	0.0327 ± 0.0010 e	0.0111 ± 0.0001 c
200331	0.0031 ± 0.0001 d	0.0049 ± 0.0006 cd	0.0347 ± 0.0003 f	0.0317 ± 0.0002 f
200113	0.0058 ± 0.0003 f	0.0058 ± 0.0003 d	0.0230 ± 0.0007 b	0.0126 ± 0.0010 d
200213	0.0018 ± 0.0001 bc	0.0015 ± 0.0001 a	0.0259 ± 0.0002 d	0.0024 ± 0.0001 a
200313	0.0017 ± 0.0001 bc	0.0055 ± 0.0001 d	0.0186 ± 0.0003 a	0.0100 ± 0.0003 b

The different lowercase letters associated with the values within the same column indicate significant difference at the level of *p* < 0.05.

**Table 5:** The structural changes of starch in recombinant flours.

Sample	Long-Range Order by XRD	Short-Range Order by FTIR		Short-Range Order by LCM-Raman	Location and Height of the Peak at 860 cm^−1^ in FTIR	
	Relative Crystallinity (%)	DO (1047/1022 cm^−1^)	DD (995/1022 cm^−1^)	FWHM of the Band at 480 cm^−1^	Wavelength (cm^−1^)	Transmittance
200115	7.22 ± 0.34 d	1.181 ± 0.001 e	1.327 ± 0.001 f	18.21 ± 0.20 d	858	0.920 ± 0.005 c
200215	7.10 ± 0.56 cd	1.271 ± 0.001 h	1.437 ± 0.002 h	15.22 ± 0.23 b	858	0.900 ± 0.004 b
200315	6.35 ± 0.21 c	1.398 ± 0.002 i	1.677 ± 0.002 i	9.83 ± 0.12 a	858	0.879 ± 0.008 a
200131	6.63 ± 0.16 c	1.136 ± 0.001 d	1.217 ± 0.001 d	16.32 ± 0.21 c	860	0.930 ± 0.002 d
200231	9.96 ± 0.56 e	1.101 ± 0.002 c	1.176 ± 0.002 c	19.87 ± 0.20 e	860	0.969 ± 0.006 e
200331	7.28 ± 0.43 d	1.081 ± 0.001 b	1.109 ± 0.001 b	15.04 ± 0.10 b	858	0.965 ± 0.002 e
200113	3.80 ± 0.55 a	1.231 ± 0.001 g	1.352 ± 0.001 g	15.46 ± 0.25 b	860	0.907 ± 0.008 bc
200213	4.70 ± 0.39 b	1.196 ± 0.002 f	1.265 ± 0.002 e	18.14 ± 0.13 d	858	0.919 ± 0.004 c
200313	3.38 ± 0.37 a	1.069 ± 0.003 a	1.103 ± 0.003 a	18.57 ± 0.18 d	856	0.973 ± 0.009 e

The different lowercase letters associated with the values within the same column indicate significant difference at the level of *p* < 0.05.

**Table 6:** The molecular order of starch by ^13^C solid-state NMR.

Sample	Double Helix (%)	Single Helix (%)	PPA (%)
200115	53.36 ± 0.04 ab	2.04 ± 0.01 d	11.02 ± 0.01 b
200215	52.20 ± 0.01 a	1.49 ± 0.02 c	11.07 ± 0.01 c
200315	59.17 ± 0.01 f	2.14 ± 0.01 d	12.16 ± 0.02 g
200131	55.47 ± 0.02 de	0.91 ± 0.31 b	10.80 ± 0.03 a
200231	56.28 ± 0.01 e	1.92 ± 0.01 d	11.67 ± 0.04 d
200331	58.19 ± 2.09 f	3.27 ± 0.38 e	11.85 ± 0.08 f
200113	54.76 ± 0.01 cd	0.09 ± 0.01 a	12.48 ± 0.01 h
200213	54.00 ± 0.02 bc	0.63 ± 0.01 b	14.90 ± 0.02 i
200313	54.84 ± 0. 11 cd	1.99 ± 0.03 d	11.75 ± 0.01 e

The different lowercase letters associated with the values within the same column indicate significant difference at the level of *p* < 0.05.

## Data Availability

The data presented in this study are available on request from the corresponding author.
